# Two Closely Related Butterflies Show Pseudo‐Concordant Patterns of Geographic Differentiation in a Heterogeneous Mountain Region

**DOI:** 10.1002/ece3.74153

**Published:** 2026-08-11

**Authors:** Luca Gregnanin, Enrico Carta, Claire Dainelli, Roberto Magnolini, Giulio Montanaro, Alessandro Grapputo, Lucio Bonato

**Affiliations:** ^1^ Dipartimento di Biologia Università di Padova Padova Italy; ^2^ National Biodiversity Future Centre Palermo Italy

**Keywords:** Alps, comparative phylogeography, *Erebia euryale*, *Erebia ligea*, genomic differentiation, morphological differentiation

## Abstract

Sympatric species are often found either to broadly match or to differ remarkably in their phylogeographic patterns. However, sometimes they show ‘phylogeographic pseudo‐concordance’, that is, they share the same geographic arrangement of population differentiation, but differ in the level of differentiation between populations. Especially in heterogeneous regions like mountains, cases of pseudo‐concordance are particularly insightful to gauge the relative role of landscape features, species traits and colonisation history in shaping evolutionary differentiation. Yet, such cases have been rarely documented so far. We investigated the differentiation patterns of two closely related butterfly species, *Erebia euryale* and *E. ligea*, in a highly heterogeneous mountain region in the south‐eastern Alps. We applied a fine‐scale geographical sampling and complemented genomic analyses (SNPs from ddRAD sequencing) with an analysis of phenotypic differentiation (morphological characters of the wings and male copulatory structures). Our findings provide strong evidence for pseudo‐concordance between the two species, which are both differentiated into two groups of populations: one occurring in the western part of the study area and the other in the eastern part, with the geographic boundary separating the two groups being coincident in the two species. However, while in *E. euryale* the two population groups correspond to lineages that appear differentiated at the species level, with negligible gene flow and significant phenotypic divergence, the two groups within *E. ligea* only show a weak genetic divergence, with substantial gene flow and no detectable phenotypic differentiation. Our case study shows how sampling populations at an adequate spatial resolution and addressing evolutionary differentiation encompassing both genomic and phenotypic components may succeed in detecting and contributing strong evidence for phylogeographic pseudo‐concordance between sympatric species in highly heterogeneous regions like mountains.

## Graphical abstract

Phylogeographic pseudo‐concordance among species—coincident spatial structuring coupled with differing levels of intraspecific differentiation—has rarely been documented in heterogeneous regions. We report a clear case in two mountain butterfly species and show how the high density of spatial sampling and the integration of phenotypic and genomic analyses are crucial to robustly detect such patterns.

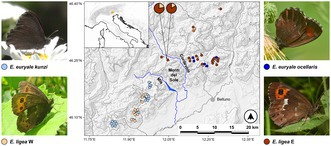

## 1 Introduction

When a species spreads over a region, both adaptive and stochastic processes lead to a variable degree of reproductive separation and geographic differentiation among populations, producing a continuum of conditions from overall panmixia and virtually null divergence to a mosaic of fully separated and divergent species (e.g., De Queiroz 2007; Seehausen et al. 2014; Zachos 2016; Stankowski and Ravinet 2021). In comparative phylogeography, a null hypothesis posits that sympatric species exhibit a similar geographic pattern of intraspecific differentiation within the shared region, a condition known as ‘phylogeographic concordance’ (Taberlet 1998; Taberlet et al. 1998; Edwards et al. 2021). Investigating concordant patterns between species has helped elucidate key processes driving population divergence in different organisms and different regions (Hewitt 2004; Gómez and Lunt 2007; Gutiérrez‐García and Vázquez‐Domínguez 2013). However, sympatric species often display spatially dissimilar patterns of differentiation within the shared region (‘phylogeographic discordance’), that is, different spatial arrangement of barriers and corridors for dispersal and gene flow between groups of populations, besides possibly different levels of differentiation between the groups. Phylogeographic discordance has been repeatedly attributed to interspecific differences in biological traits (such as dispersal ability, ecological niche and life history; Hodges et al. 2007; Beavis et al. 2011; Papadopoulou and Knowles 2015, 2016; Kato et al. 2025) and/or to interspecific differences in the colonisation histories (e.g., timing of colonisation events or of intraspecific differentiation; Soltis et al. 2006; Carstens and Richards 2007; Leaché et al. 2007), or to stochastic population dynamics (Kato et al. 2025).

An alternative scenario is the so‐called ‘phylogeographic pseudo‐concordance’, acknowledged when the phylogeographic structures of two or more sympatric species coincide in the spatial arrangement of barriers and corridors, yet differ in the magnitude of evolutionary differentiation between population groups (Soltis et al. 2006; Avise 2009). When looking for pseudo‐concordance, the spatial pattern of differentiation should be assessed at a spatial scale appropriate to the dispersal abilities of the organisms and to the regional density of potential barriers (e.g., Hodges et al. 2007). Moreover, the magnitude of evolutionary differentiation between populations can be thoroughly compared across species only with a joint investigation of multiple biological features (e.g., genomic, phenotypic, physiological and ecological features) within an integrative framework, because different features frequently display mismatched geographic patterns of differentiation (e.g., mito‐nuclear discordances, cases in which conservative morphology conceals cryptic genetic divergence, etc.; De Queiroz 2007; Schlick‐Steiner et al. 2010).

Pseudo‐concordance is expected to be more probable in environmentally uniform regions, where the very few environmental discontinuities and gradients are expected to set the same barriers and corridors for several species, even though the levels of differentiation may vary between species. Indeed, comparative phylogeographic studies in relatively simple geographical settings frequently reveal patterns that may be acknowledged as pseudo‐concordant (e.g., Oaks 2019; Gottscho et al. 2025). Instead, pseudo‐concordance is expected to be less frequent across environmentally complex settings, like mountain regions, where the presence of many environmental discontinuities and gradients determines a higher chance of different barriers or corridors for different species. Indeed, cases of pseudo‐concordance across highly heterogeneous regions have been rarely documented (e.g., Soltis et al. 2006; Garrick et al. 2012; Busschau et al. 2022), although this may be partially explained also by the fact that they require a relatively more abundant and denser spatial sampling across several and closely spaced potential barriers.

Nonetheless, pseudo‐concordance in environmentally complex regions is of great interest in evolutionary biology because it may provide insights into the role of the landscape in shaping and constraining the geographic differentiation of a species, besides the role of the biological traits and colonisation history of the species. Therefore, searching for additional cases of pseudo‐concordance in highly heterogeneous regions appears useful to collect evidence to gauge the relative interplay of geographical, biological and historical factors in moulding intraspecific evolutionary differentiation and speciation.

We report on a case of pseudo‐concordance between two butterfly species, *Erebia euryale* and *E. ligea*, across a remarkably heterogeneous mountain region in the south‐eastern Alps. We compared the two geographical patterns of evolutionary differentiation by applying a fine‐scale geographical sampling (appropriate for the dispersal abilities of the animals and the heterogeneity of the landscape) and by analysing both genomic data (obtained through ddRAD sequencing) and morphological characters of primary ecological and evolutionary significance (wings and copulatory structures).

## 2 Material and Methods

### 2.1 Studied Species

*Erebia euryale* and *E. ligea* inhabit the major mountain chains of the Western Palearctic (Kudrna et al. 2011), where they are frequently found in syntopy (e.g., Sonderegger 2005; pers. obs.). Throughout the Alps, they are mainly found in woodland clearings (*E. euryale* also in marginal meadows and pastures), in broad and largely overlapping altitudinal ranges, usually between 700 and 2500 m for *E. euryale* and between 500 and 2000 a.s.l. for *E. ligea* (Sonderegger 2005; Verovnik et al. 2012; Bonato et al. 2014).

Even though they have been consistently identified as distinct (yet very closely related) species in the most recent phylogenetic analyses (Peña et al. 2015; Wiemers et al. 2020; Klečková et al. 2023; Augustijnen et al. 2024) and their species‐level separation is universally accepted, *E. euryale* and *E. ligea* are very similar in many respects. They have the same karyotype (Augustijnen et al. 2024) and share haplotypes of the mitochondrial barcode region (Dincă et al. 2021; Dapporto et al. 2022). They are also very similar in colour pattern of wings and shape of copulatory structures: only a few subtle differences have been proposed as diagnostic characters (Verity 1953; Dincă et al. 2011), and many characters show a broad range of intraspecific variation (Cupedo 2010). The two species also largely overlap in many key life history traits (Middleton‐Welling et al. 2020), such as phenology (Sonderegger 2005; Zakharova 2010; Klečková et al. 2014), habitat preferences (Klečková et al. 2014) and trophic ecology (Sonderegger 2005; Clarke 2022).

Dispersal abilities have not been investigated quantitatively in *E. euryale* and *E. ligea*; however, in this respect, the two species are expected to be similar to each other and to other ecologically similar and often syntopic *Erebia* species: capture‐mark‐recapture studies conducted on *E. aethiops* , *E. epiphron*, *E. pronoe* and *E. sudetica* in comparable mountain environments indicate that adults rarely shift beyond 1 km during their lifetime (Kuras et al. 2003; Slamova et al. 2013; Wendt et al. 2021; Gunson et al. 2023).

### 2.2 Study Area

**FIGURE 1:**
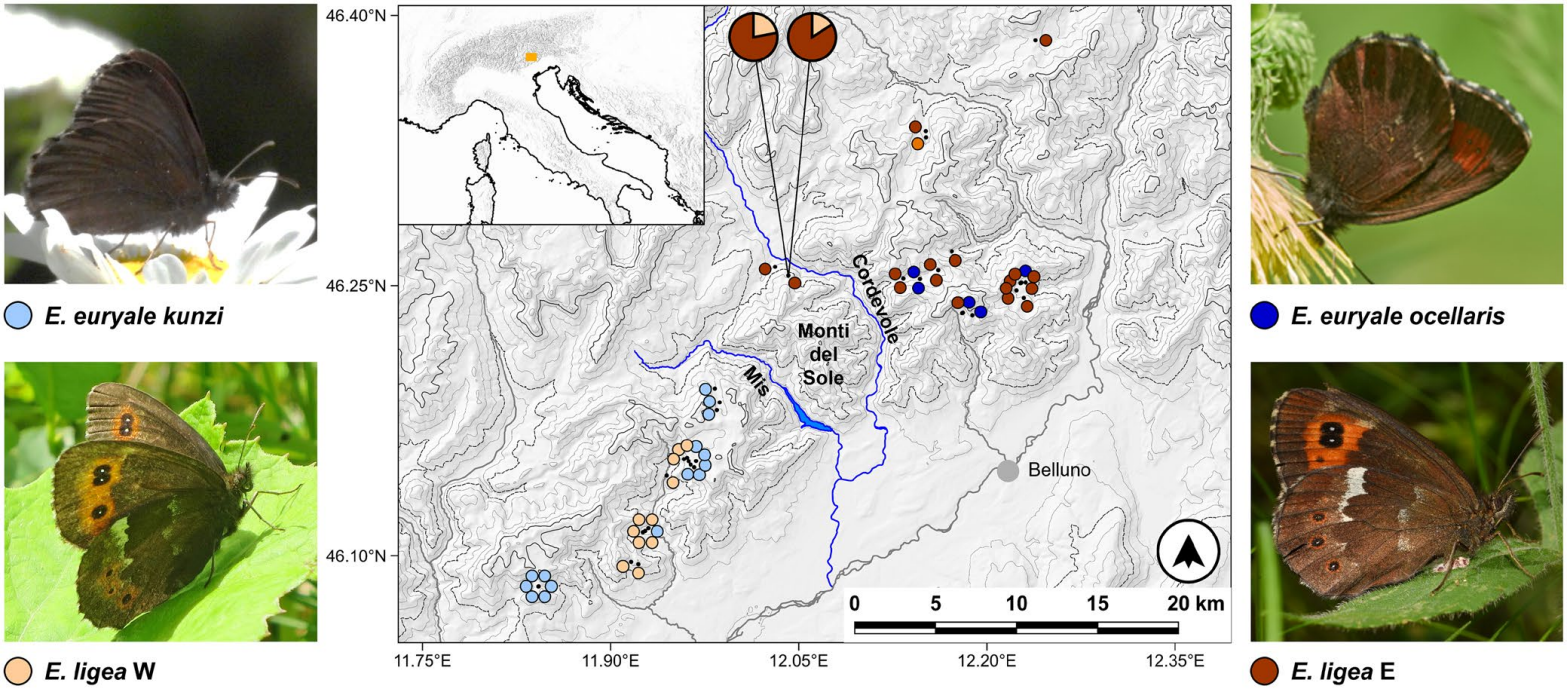
Geographic distribution of the sampled specimens of *E. euryale* and *E. ligea* in the study area. Black points: Sampling plots. Coloured circles: Assignment of unadmixed specimens (STRUCTURE ancestry coefficients > 99%) to the groups resulting from the STRUCTURE analysis of the *reference‐based* assembled datasets of SNPs with < 10% missing data (see Figure 2). Pie charts: Ancestry of two admixed *E. ligea* specimens. Contour lines: Elevation contours at 250 m intervals (light grey), and 1000 m intervals (dark grey: Dashed for 1000 m and solid for 2000). Photos of representative specimens of the population groups: *E. euryale kunzi* from Vette Feltrine, 12.VII.2023, photo E.C.; *E. euryale ocellaris* from Val Vescovà, 18.VII.2024, photo L.G.; *E. ligea* W, from Val Noana, 04.VIII.2019, photo Sandra Rossa; *E. ligea* E, from Cajada, 7.VII.2026, photo L.B.

We investigated an area of about 900 km^2^ in the Southern Dolomites, located in the south‐eastern Alps (Figure 1) and mostly comprised within a protected area (Parco Nazionale delle Dolomiti Bellunesi). This area has a complex topography, with elevation between 300 m and 2500 m, and therefore includes the entire elevational ranges known for *E. euryale* and *E. ligea* in the Alps (see Section 2.1). It encompasses a major mountain range clearly bounded to the south, east and west by large and deep valleys, but maintaining continuity with other Dolomites massifs to the north. The mountain range is crossed by two deep, narrow valleys, Cordevole and Mis, which run predominantly north–south and delimit a central massif known as Monti del Sole (Figure 1).

The study area is inhabited by both *E. euryale* and *E. ligea*, which frequently occur syntopically (pers. obs.). Based on phenotypic and genetic evidence, *E. euryale* populations in this region are currently assigned to two subspecies, *E. e. kunzi* and *E. e ocellaris*, which are thought to substitute each other from south‐west to north‐east (Cupedo 2010; Cupedo and Doorenweerd 2022). Conversely, all populations of *E. ligea* inhabiting the study area and the surrounding regions are considered undifferentiated and assigned to a single subspecies (Verity 1953).

### 2.3 Sampling of Specimens

**FIGURE A1:**
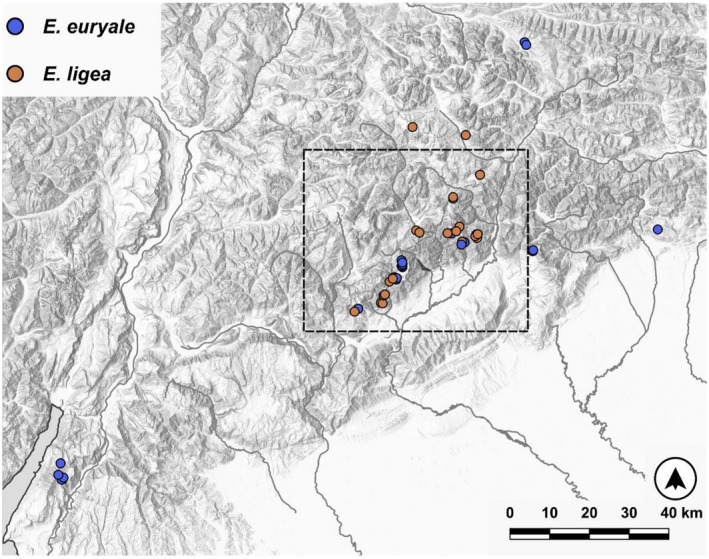
Geographic distribution of all specimens of *E. euryale* and *E. ligea* included in the analyses. The dashed rectangle includes the study area (Figure 1).

We sampled 33 adult specimens of *E. euryale* and 35 of *E. ligea* from 36 plots within the study area (Figure 1), and 18 more specimens of either species from other localities of the south‐eastern Alps outside the study area (Table A1, Figure A1). To serve as outgroups in the phylogenetic analyses (see Section 2.8), we also sampled one specimen of *E. cassioides* and one of *E. gorge* (Table A1).

All specimens were collected between June and August of 2022 and 2023. They were captured with a butterfly net, handled carefully to minimise wing damage and scale abrasion (to allow for subsequent morphological analyses; see Section 2.9) and euthanized using either ethyl acetate vapours or the compression of the thorax. The specimens were kept at ambient temperature while in the field (for no more than 3 days), then stored at −20°C until DNA extraction. Species identification was confirmed in the laboratory based on the wing characters considered the most diagnostic, primarily the pattern of male androconial scales (Warren 1936; Verity 1953; Sonderegger 2005). Specimens of *E. euryale* were tentatively assigned to subspecies based on the diagnostic characters and the geographical ranges described by Cupedo (2010, 2014) and Cupedo and Doorenweerd (2022). All specimens are preserved at the Department of Biology, University of Padova.

### 2.4 DNA Extraction and Sequencing

DNA was extracted from the thorax and/or the abdomen of each specimen using the Blood & Tissue Kit (Qiagen), following the manufacturer's instructions. DNA concentration was measured via fluorimetry using a Qubit 4.0 fluorometer (Life Technologies) while DNA purity was assessed based on the A260/280 and A260/230 absorbance ratios using a Nanodrop spectrophotometer (Thermo Fisher Scientific Inc.). The extracted DNA was then outsourced to IGATech (Udine, Italy) for ddRAD library preparation and sequencing. Libraries were prepared mostly following Peterson et al. (2012): 300 ng of DNA from each specimen were double‐digested with 2.4 U of both *NspI* and *MboI*. Fragments were ligated to 2.5 pmol of overhang barcoded adapters on both cut sites with 160 U of T4 DNA ligase (New England BioLabs). Libraries were pooled and purified with 1.5 volumes of AMPureXP beads (Agencourt), and fragments of 480–600 bp were collected with a BluePippin instrument (Sage Science Inc.). The gel‐eluted fraction was amplified with indexed primers using the Phusion High‐Fidelity PCR Master Mix (New England BioLabs). Libraries were sequenced with a NovaSeq 6000 sequencer (Illumina) in paired‐end mode, with 150 cycles, following the manufacturer's instructions.

### 2.5 Processing of DNA Sequences

Raw reads were demultiplexed with process_radtags (v. 2.66), in the Stacks pipeline (Catchen et al. 2013). We removed the reads with uncalled bases, or with raw Phred score < 10 and rescued RAD‐Tag cut sites (options −c, −q and −r of process_radtags, respectively). All other parameters were set as default.

After excluding specimens with < 1 million reads, we assembled the ddRAD loci both with a *reference‐based* and a *de novo* approach (Catchen et al. 2013).

For the *reference‐based* assembly we used the genome sequence of a specimen of *Erebia ligea* from the Carpathians (GenBank accession: GCA_917051295.2; Lohse et al. 2022). We used bwa (v. 0.7.17; Li and Durbin 2009) with default parameters for the alignment of the reads to the genome, and the ref_map.pl pipeline of Stacks (v. 2.66) with default parameters for the assembly of the loci and SNP calling.

For the *de novo* assembly we used Stacks (v. 2.65), optimising the three parameters *M*, *m* (ustacks) and *n* (cstacks) by maximising the number of polymorphic loci present in ≥ 80% of specimens (‘R80’ method; Paris et al. 2017). Due to computational constraints, we optimised the parameters using a subset of 17 specimens of *E. euryale* and *E. ligea* (Table A1), chosen to represent the variation of the number of filtered reads, of the wing colour pattern and of the geographic provenance. We used the R package RADstackshelpR (v. 0.1.0; DeRaad 2021) to compare the number of polymorphic loci retained with each combination of parameters and to select the optimal value for each parameter. We first evaluated values of *m* between 3 and 7, fixing *M* and *n* to their default values (*M* = 2, *n* = 1) and then values of *M* between 1 and 8, fixing *m* and *n* to their default values (*m* = 3, *n* = 1). As we obtained optimal values of *m* = 7 and *M* = 7, we then evaluated values of *n* between 6 and 8 (i.e., the optimal value of *M* ± 1), setting values of *m* and *M* to their optimal values, obtaining an optimal value of *n* = 8.

### 2.6 Genetic Population Structure Analysis

We first investigated the genetic population structure of the two species, analysing all the specimens with > 1 million reads (see Section 2.5), also considering those sampled outside the study area.

We used STRUCTURE (v. 2.3.4; Pritchard et al. 2000) automated with StrAuto (v. 1.0; Chhatre and Emerson 2017), with *K* = 1–8 (higher values were hindered by computational constraints). We ran the analyses with 10 replicates for each *K* value, with 1.5 million iterations after a burn‐in of 10,000 iterations. We used an admixture model with correlated allele frequencies and without prior information on sampling plots. All other parameters were set as default. We compared the results of different replicates using Clumpak (Kopelman et al. 2015), assessing the support for different values of *K* through the log‐probability (calculated as described in STRUCTURE's manual) and its second‐order rate of change among successive values of *K* (ΔK; Evanno et al. 2005). For each specimen, we averaged the ancestry coefficients among all replicates, excluding single replicates only when producing a partition of the specimens different from the prevailing one (Kopelman et al. 2015).

Analyses with STRUCTURE were repeated on different subsets of the *reference‐based* and *de novo* assemblies, with different amounts of missing data (retaining the SNPs present in at least 70%, 80%, 90% or 100% of the specimens). We retained only one randomly chosen SNP for each ddRAD locus. We did not exclude SNPs with allele frequencies departing from the Hardy–Weinberg equilibrium. Since the results of these analyses were largely consistent (see Results: Section 3.1), we only carried out the subsequent analyses on datasets obtained with the *reference‐based* assembly (which included a larger number of SNPs) and retaining SNPs present in at least 90% of the specimens. In particular, in order to investigate further differentiation, we ran similar analyses separately for each of the groups recovered by the first analysis, with the same settings.

We also investigated the population genetic structure of the two species with a Discriminant Analysis of Principal Components (DAPC; Jombart et al. 2010), carried out with the R package adegenet (v. 2.1.10; Jombart 2008) on the same sample analysed with STRUCTURE. We first performed a Principal Component Analysis on the SNP dataset to obtain a reduced set of uncorrelated variables and then clustered the specimens through sequential *k*‐means based on all the principal components (PCs). We considered all values of *k* between 1 and 15 and selected the best fitting *k* according to the Bayesian Information Criterion (BIC). We finally ran a Discriminant Analysis of the *k* groups on the first 50 PCs, accounting for approximately 90% of the initial variation, keeping all the linear discriminants axes. We repeated the analysis with other values of *k* with sub‐optimal but similar values of BIC and supported by the analyses with STRUCTURE.

### 2.7 Estimation of Genetic Differentiation and Migration Rates

To compare the degree of genetic differentiation between the groups identified within each species, we calculated F_ST_ (Weir and Cockerham 1984) between pairs of population groups within each species and between the two species, using the R package assigner (v. 0.5.8; Gosselin et al. 2020). Confidence intervals (95%) were estimated through 1000 bootstrap replicates.

Since the pairwise F_ST_ is a relative measure of the genetic differentiation between populations and is influenced by the genetic diversity within each population, we also calculated d_XY_ (Nei and Li 1979), which estimates the absolute genetic divergence between populations (Burri 2017), using pixy (v. 2.0.0.beta14; Korunes and Samuk 2021). We used a window size of 100,000 nucleotides and subsequently aggregated the estimates per window to obtain a ‘genome‐wide’ estimate.

To assess the recent gene flow between the groups within each species, we estimated the average proportion of migrants over the past few generations, using BAYESASS (Wilson and Rannala 2003), optimised for SNP data in the software BA3‐SNPs (v. 1.0.0; Mussmann et al. 2019). In particular, we used BA3‐SNPs‐autotune (Mussmann et al. 2019) to optimise the ‘mixing parameters’ for migration rates (*M* = 0.2125), allele frequencies (*A* = 0.3250) and inbreeding coefficients (*F* = 0.1000). The mixing parameters control the magnitude of change in each variable between successive iterations of the Markov chains. We ran BA3‐SNPs for 20 million iterations, following a burn‐in of 2 million iterations, using the same dataset employed for estimating genetic differentiation. We assessed convergence of the MCMC with Tracer (v. 1.7.2; Rambaut et al. 2018).

Genetic differentiation, genetic divergence, and migration rates were estimated from a concatenation of SNPs, only considering the specimens within the study area and only for population groups represented by at least five specimens. Since the d_XY_ values were computed only from shared variant sites, they are informative for comparing the two species but cannot be interpreted as absolute estimates of divergence (Korunes and Samuk 2021).

### 2.8 Phylogenetic Analyses

To assess whether the groups of populations identified through the population genetic analyses were reciprocally monophyletic and to estimate their phylogenetic relationships, we performed a Maximum Likelihood analysis using IQ‐TREE (v. 2.3.4; Minh et al. 2020), including all specimens of *E. euryale* and *E. ligea*, along with two outgroup specimens ( *E. cassioides* and *E. gorge*; Table A1). The best‐fitting substitution model was determined using ModelFinder (Kalyaanamoorthy et al. 2017) implemented in IQ‐TREE, based on BIC. Only models corrected for the ascertainment bias were considered, as recommended for datasets without constant characters (Lewis 2001), such as SNP datasets. We estimated branch supports using 10,000 ultrafast bootstrap replicates (Hoang et al. 2018). We carried out the analyses on two SNP datasets: (i) one in which heterozygous sites were encoded using IUPAC ambiguity codes, and (ii) one in which a single allele was randomly sampled for each heterozygous site.

### 2.9 Analysis of Morphological Differentiation

**FIGURE A2:**
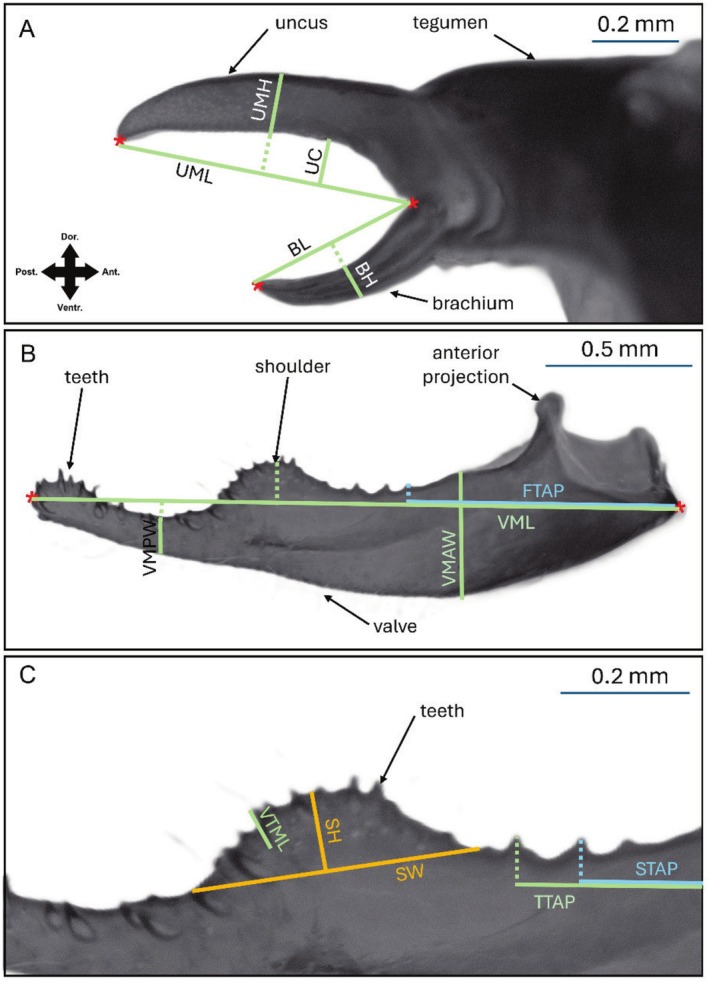
Examples of standard photos and measurements taken on male copulatory structures (specimen of *E. euryale*: PD‐R 975). A: Right side of the dorsal copulatory structures; B: External side of the right valve; C: Intermediate part of the dorsal margin of the right valve. Abbreviations as in Table A2. Asterisks: See the protocol above.

To evaluate the extent of morphological differentiation between the genetic groups identified within each species, we examined the wings and the male copulatory structures. Accordingly, the analyses were restricted to male specimens. For both the wings and the copulatory structures, we considered all morphological characters previously proposed to distinguish the two species (*E. euryale* and *E. ligea*) and their subspecies occurring in the south‐eastern Alps (Warren 1936; Heydemann 1943; Verity 1953; Cupedo 2010; Bouaouina et al. 2023). Specifically, we defined 16 wing characters (describing size, androconial scales and colour pattern; Table A2) and 24 genital characters (describing size and shape of the uncus, brachia and valves; Table A2, Figure A2). They include categorical, meristic and continuous characters (linear measurements). Whenever possible, we followed the operational definitions of the characters proposed by Cupedo (2010). Specimens were examined in a random order and blindly with respect to both the sampling plot and the genetic group.

All wing characters were assessed by a single author (E.C.) on all male specimens sampled from the study area with well‐preserved wings (Table A1).

The copulatory structures were dissected by digesting the posterior third of the abdomen of each male specimen in 10% KOH for 6–18 h at room temperature and then rinsing it with water to remove KOH. The valves were isolated from the dorsal copulatory structures (uncus, brachia and tegumen) with fine needles, cleaned and stored in glycerol at room temperature. For each specimen, standardised digital photos of the dorsal copulatory structures and one of the valves (preferentially the right one whenever sufficiently well preserved) were taken using a Leica Flexacam C5 mounted on a Leica MCZ stereomicroscope with a 2.5× objective lens, following a protocol described in the Appendix.

All characters of the copulatory structures were measured on the aforementioned photos. Linear measurements were taken using imageJ (v. 1.53; Schneider et al. 2012). Most of the characters were measured by a single author (L.G.) across all the male specimens sampled within the study area with sufficiently well‐preserved copulatory structures (Table A1). However, as the evaluation of one character (describing the arrangement of teeth on the valves; see Table A2) was hard to standardise, this character was independently assessed by multiple authors (L.B., L.G., G.M.) on all specimens, and their evaluations were subsequently averaged.

To assess the overall pattern of morphological variation, we performed a Nonmetric Multidimensional Scaling (NMDS) on 3 dimensions, using the R package vegan (v. 2.6; Oksanen et al. 2022). We carried out a joint analysis with all characters, as well as separate analyses for wings and copulatory structures. Given that the datasets included a mixture of categorical, meristic and continuous characters (Table A2), we carried out the NMDS on Gower's distances between the specimens (Gower 1971). To test whether genetic groups differed significantly in morphological characters, we carried out a PERMANOVA on the Gower's distances, using the R package vegan and pairwise post hoc PERMANOVA tests, using the R package pairwiseAdonis (v. 0.4.1; Martinez Arbizu 2017).

All the analyses with R were performed using version 4.3.1 (R Core Team 2023).

## 3 Results

### 3.1 Genetic Population Structure

The ddRAD sequencing of 47 specimens of *E. euryale*, 39 specimens of *E. ligea*, and two outgroup specimens produced a total of 415 million reads (ranging between 19,000 and 11 million reads for each specimen of either *E*. *euryale* or *E*. *ligea*; Table A1). After excluding 16 specimens for which we obtained less than 1 million reads and one specimen whose library was most likely affected by contamination, we assembled a dataset comprising 34 specimens of *E. euryale* (21 specimens from the study area) and 35 specimens of *E. ligea* (31 specimens from the study area), with on average 5.6 million reads for each specimen, plus the 2 outgroup specimens (Table A1).

**FIGURE 2:**
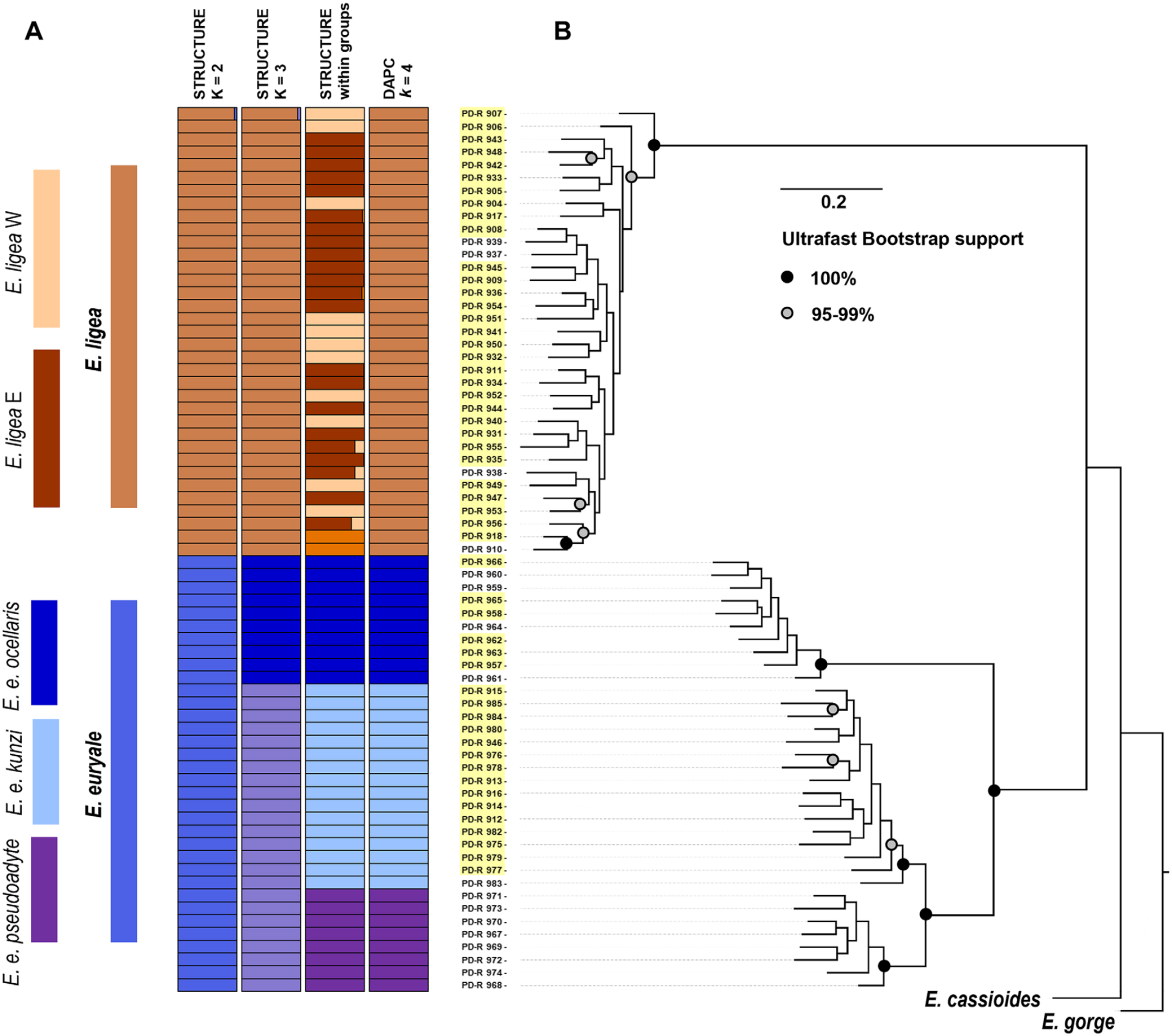
Results of population structure analyses and phylogenetic analyses on *E. euryale* and *E. ligea* in the south‐eastern Alps. A: Ancestry coefficients estimated by STRUCTURE (for all 69 specimens for *K* = 2 and *K* = 3 and within each of the groups found with *K* = 3) and assignment probabilities obtained by the DAPC with *k* = 4. B: Maximum Likelihood phylogenetic tree inferred with IQ‐TREE from 1168 SNPs (with one allele randomly selected for each heterozygous genotype). Specimens from the study area (Figure 1) are highlighted in yellow.

**FIGURE A3:**
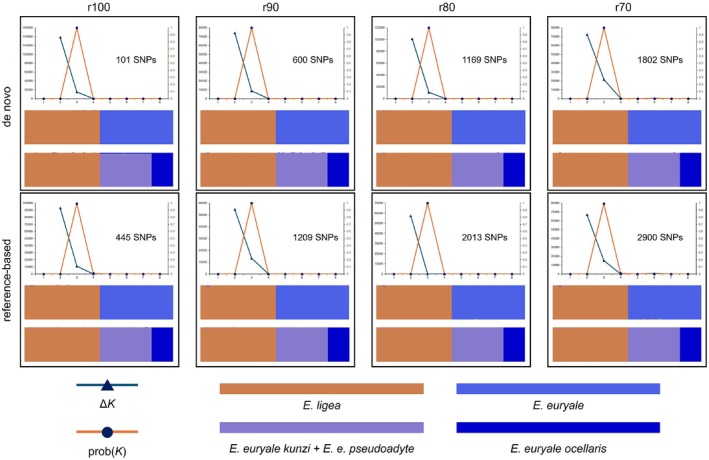
Comparison of results of STRUCTURE across alternative assembly methods and different proportions of missing data. Top panels: Supports for different *K* values; bottom panels: Barplots of ancestry coefficients estimated for the specimens under the *K* most supported by ΔK (top) and prob.(*K*) (bottom).

Population structure analyses conducted using STRUCTURE on all 69 specimens of the two species produced largely consistent results: across all analyses, *K* = 2 had the highest ΔK, while *K* = 3 had the highest log‐probability (Figure A3). At *K* = 2, STRUCTURE consistently distinguished all *E. euryale* specimens (with ≥ 99% ancestry) from all *E. ligea* specimens (with ≥ 95% ancestry) (Figure 2A). At *K* = 3, the analyses further separated the *E. euryale* specimens referred to the subspecies *E. e. ocellaris* (with ≥ 99% ancestry) from all other *E. euryale* specimens (with ≥ 99% ancestry) (Figure 2A). All specimens of *E. e. ocellaris* from the study area were collected east of the Monti del Sole and the Cordevole valley (Figure 1).

**FIGURE A4:**
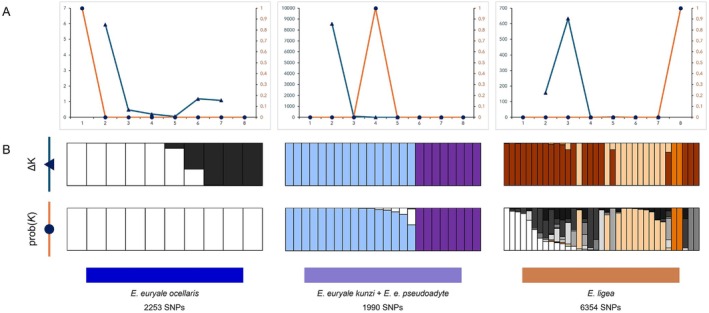
Results of the STRUCTURE analyses within the genetic groups identified in the STRUCTURE analyses on all specimens. A: ΔK and prob.(*K*) supports for different values of *K*. B: Barplots of the corresponding ancestry coefficients estimated by STRUCTURE assuming the best fitting *K* according to ΔK and prob.(*K*). Colours as in Figures 1 and 2, with additional grey shades representing groups not shown in other figures. As ΔK is defined only for *K* ≥ 2, it can not discriminate between *K* = 1 and *K* = 2.

The analysis performed with STRUCTURE only including the *E. e. ocellaris* specimens revealed no further genetic differentiation (Figure 2A, Figure A4). In contrast, the analysis on the remaining *E. euryale* specimens separated all the specimens of *E. e. kunzi* from other specimens sampled outside the study area and recognisable as belonging to a different subspecies not present in the study area (*E. e. pseudoadyte*, see Cupedo 2010) (in both cases with ≥ 99% ancestry) (Figure 2A). Notably, all specimens of *E. e. kunzi* from the study area were collected west of the Monti del Sole and the Mis valley and (Figure 1).

The analysis performed with STRUCTURE on *E. ligea* alone similarly separated the specimens collected west of the Monti del Sole (assigned to one group with ≥ 99% ancestry, hereafter *E. ligea* W) from those collected east of Mis valley (most of them assigned to a separate group with ≥ 96% ancestry, hereafter *E. ligea* E) (Figure 1, Figure 2A). However, two specimens collected on the eastern slopes of the Monti del Sole, along the Cordevole valley, were assigned to *E. ligea* E with lower ancestry coefficients (78%–84%) (Figure 1). Additionally, a specimen from the eastern part of the study area was assigned to a third group with ≥ 99% ancestry, together with another specimen collected north of the study area (Figure 1, Figure 2A, Figure A4).

**FIGURE A5:**
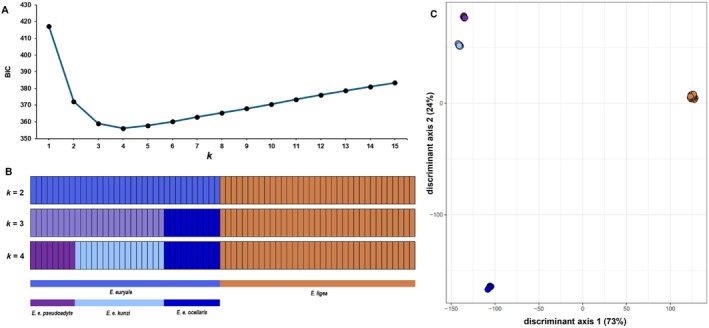
Results of the Discriminant Analysis of Principal Components. A: BIC support for different numbers of groups (*k*). B: Assignment probabilities of each specimen for different values of *k*. C: Scatterplot of the coordinates of the specimens in the first two linear discriminant axes.

The Discriminant Analysis of Principal Components (DAPC) grouped the specimens into four clusters, each corresponding to genetic groups already identified by STRUCTURE: *E. e. ocellaris*, *E. e. kunzi*, *E. e. pseudoadyte* and *E. ligea* (Figure 2A, Figure A5). The first discriminant axis (accounting for 73% of between‐group discriminatory variance) separated the specimens of *E. euryale* from those of *E. ligea*, while the second axis (accounting for 24% of variance) distinguished among the three *E. euryale* groups, with *E. e. ocellaris* positioned furthest from *E. e. kunzi* and *E. e. pseudoadyte*. Suboptimal partitions separated only *E. euryale* and *E. ligea* at *k* = 2 and also *E. e. ocellaris* from the remaining *E. euryale* at *k* = 3. No additional clear geographic structure was detected at *k* > 4.

### 3.2 Genetic Differentiation and Migration Rates

**FIGURE 3:**
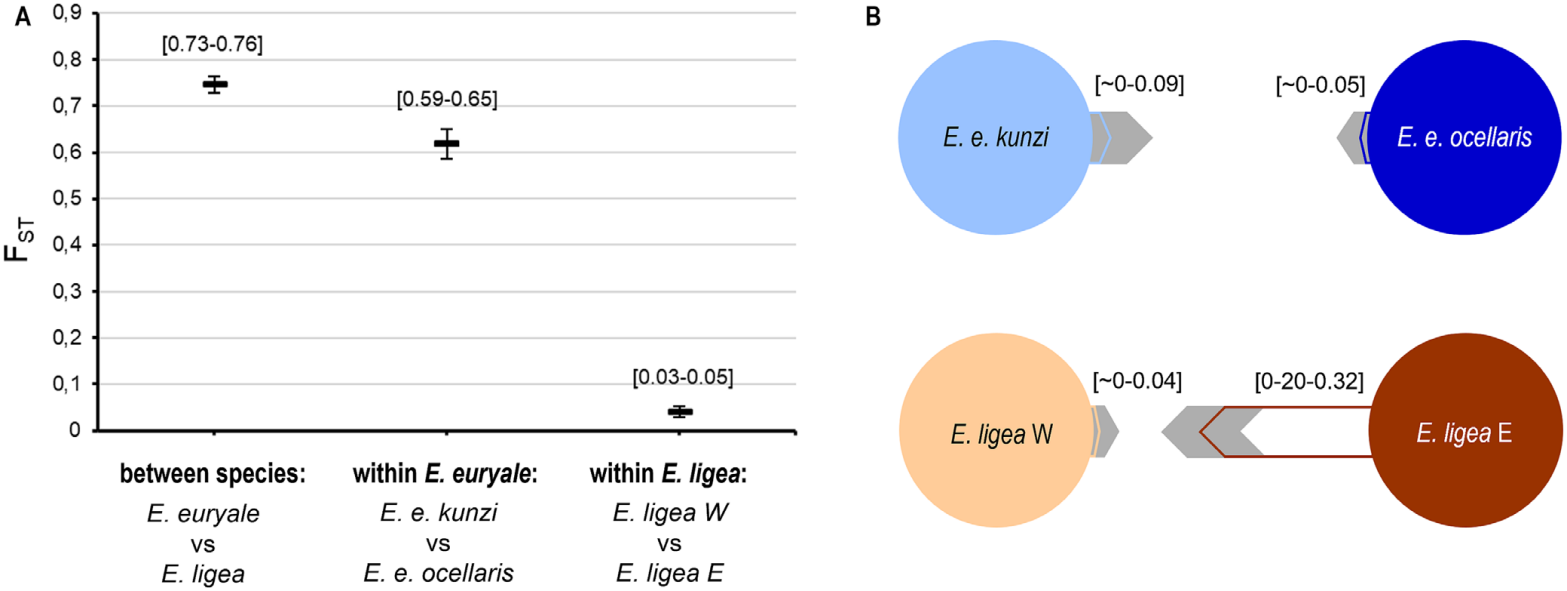
Genetic differentiation and migration rates between population groups estimated for 52 individuals of *Erebia euryale* and *E. ligea* from the study area. A: Pairwise F_ST_ values between *E. euryale* and *E. ligea* and between the two genetic groups within each species, based on 1401 SNPs (whiskers: 95% confidence intervals). B: Recent migration rates (m) between the two genetic groups within each species, estimated based on 1412 SNPs (grey arrows: 95% Highest Posterior Density intervals).

Within the study area, the genetic differentiation and divergence between the two groups of *Erebia euryale* (*E. e. kunzi* and *E. e. ocellaris*; F_ST_ = 0.62; d_XY_ = 0.33) were significantly higher than those between the two main groups of *E. ligea* (*E. ligea* W and *E. ligea* E; F_ST_ = 0.04; d_XY_ = 0.10; Figure 3A). Moreover, the differentiation between *E. e. kunzi* and *E. e. ocellaris* was only slightly lower than that between the two species *E. euryale* and *E. ligea* (F_ST_ = 0.75; Figure 3A).

Recent migration rates estimated with BAYESASS revealed very low levels of gene flow between *E. e. kunzi* and *E. e. ocellaris* (Figure 3B), but substantial gene flow between *E. ligea* groups at least in one direction (Figure 3B).

### 3.3 Phylogenetic Relationships

**FIGURE A6:**
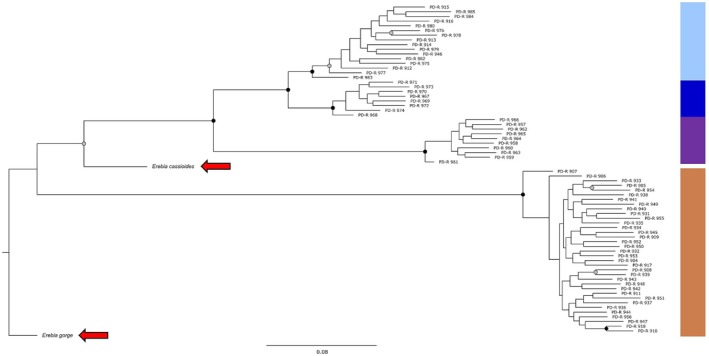
Maximum likelihood phylogenetic tree obtained with IQTREE on 955 SNPs (including heterozygous sites, which were coded with IUPAC ambiguity codes). The two red arrows indicate the two outgroup specimens. Note that the number of SNPs is slightly smaller than in the analysis with randomly selected alleles (see Figure 2), since the use of ambiguity codes made some of the sites invariant (a condition preventing the use of the correction for ascertainment bias; see Section 2.8), these sites were therefore excluded from the analysis.

All Maximum Likelihood phylogenetic analyses, carried out using the best fitting substitution model (TVM + F + ASC + G4), resolved both *E. euryale* and *E. ligea* as monophyletic (Figure 2B, Figure A6). Also, each of the three genetic groups of *E. euryale* (belonging to the subspecies *E. e. ocellaris*, *E. e. kunzi* and *E. e. pseudoadyte*) were found reciprocally monophyletic (Figure 2B, Figure A6), with full bootstrap support, and *E. e. kunzi* and *E. e. pseudoadyte* were recovered as sister groups (Figure 2B, Figure A6). In contrast, most of the internal nodes within *E. ligea* were poorly supported and the two main groups identified by the population structure analyses were not recovered as monophyletic (Figure 2B, Figure A6).

### 3.4 Morphological Differentiation

**FIGURE 4:**
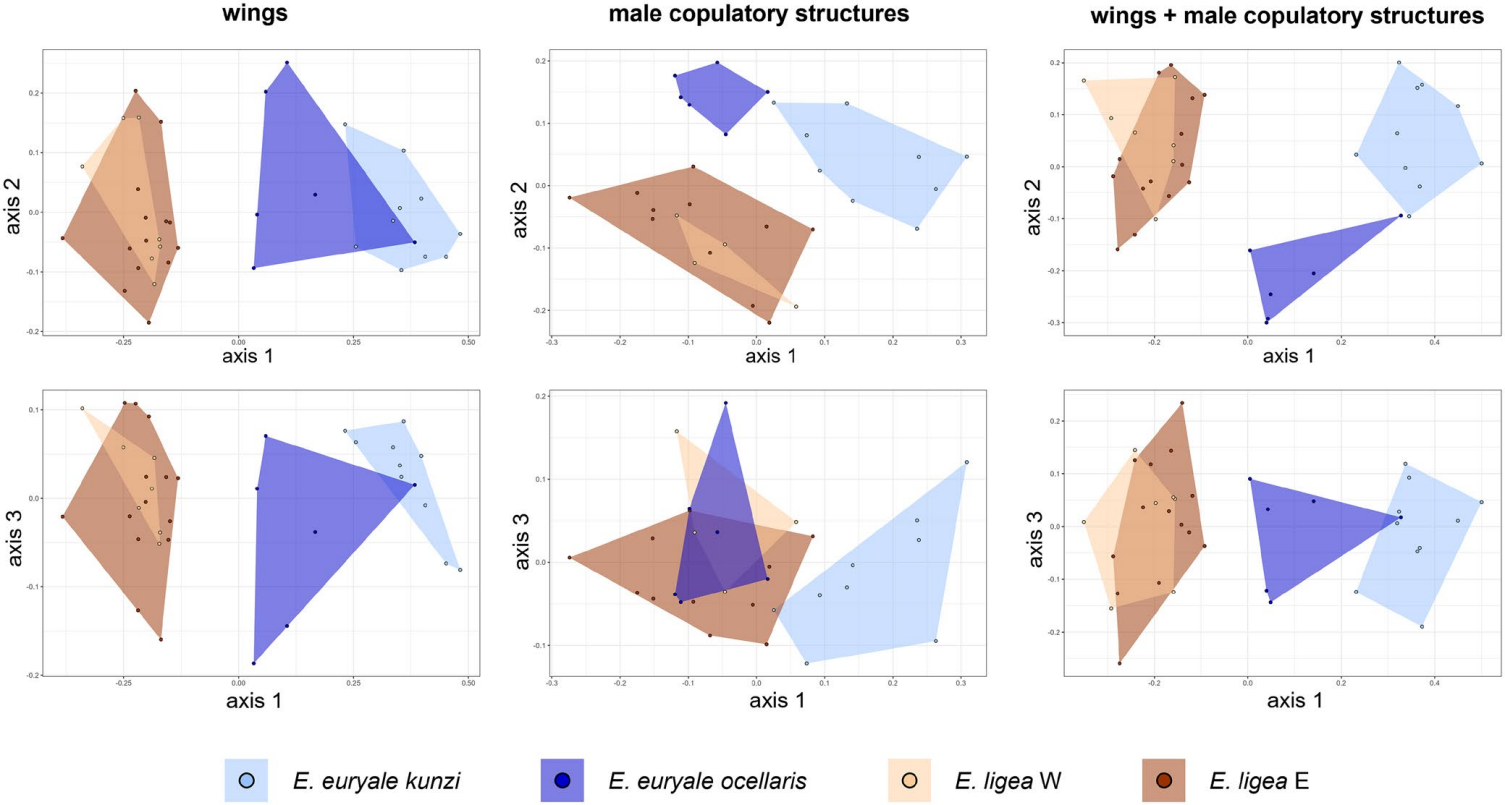
Ordination of the male specimens of *Erebia euryale* and *E. ligea* from the study area along three axes of Nonmetric Multidimensional Scaling (NMDS). Analyses were performed on wing characters (*n* = 37 specimens), copulatory characters (*n* = 30) and combined morphological characters (*n* = 37).

The NMDS based on morphological characters successfully ordinated male specimens from the study area across three dimensions (Figure 4), both when considering all characters jointly (*n* = 37 specimens; stress value = 0.10) and when analysing wings and copulatory structures separately (*n* = 37 and 30 specimens, stress values = 0.06 and 0.09, respectively).

Across all analyses, *E. e. kunzi* and *E. e. ocellaris* showed little to no overlap in any of the three NMDS dimensions. These two groups were significantly different in both wings and male copulatory structures (pairwise PERMANOVA: *p* = 0.002 for wings, *p* = 0.001 for copulatory structures, *p* = 0.002 for all characters analysed jointly). In contrast, *E. ligea* W and *E. ligea* E showed substantial overlap across all three dimensions and did not differ significantly (pairwise PERMANOVA: *p* = 0.302 for wings, *p* = 0.121 for copulatory structures, *p* = 0.305 for all characters analysed jointly).

## 4 Discussion

### 4.1 Pseudo‐Concordance Between *E. euryale* and *E. ligea* Across the Southern Dolomites

Our results indicate that the patterns of geographic differentiation of *E. euryale* and *E. ligea* in the Southern Dolomites are ‘pseudo‐concordant’ (Soltis et al. 2006): both species are structured into two main population groups and in both species the two groups substitute each other across the same narrow zone (Figure 1); however, the levels of differentiation between the two groups are dissimilar between *E. euryale* and *E. ligea* (Figure 2, Figure 3, Figure 4).

Our analyses on the sampled specimens allow us to locate the substitution zone within both species somewhere between the Mis and Cordevole valleys, that is, within an area ca. 10 km wide corresponding to the Monti del Sole massif (Figure 1).

Several lines of evidence indicate that the two population groups of *E. euryale* inhabiting the study area (recognised as belonging to the subspecies *E. e. kunzi* and *E. e. ocellaris*; Cupedo 2010) are in a very advanced stage of the speciation process: (i) the two groups are genetically distinct according to all population‐structure analyses (Figure 2); (ii) their genetic differentiation is high, with F_ST_ values only slightly lower than F_ST_ between the two species *E. euryale* and *E. ligea* (Figure 3) and comparable to values reported for other parapatric *Erebia* lineages that are either recognised as species or considered subspecies with substantial reproductive isolation at contact zones (e.g., Bouaouina et al. 2023; Jospin et al. 2023; Augustijnen and Lucek 2024); (iii) recent migration rates between the two population groups are negligible (Figure 3) and similar to those estimated between other *Erebia* lineages considered separate species (Jospin et al. 2023); (iv) both groups are reciprocally monophyletic with strong support in all phylogenetic analyses (Figure 2); (v) males of the two groups differ slightly but significantly in average wing size and colour patterns and in the average size, overall shape and some details of the copulatory structures (Figure 4). In contrast with *E. euryale*, the differentiation between the two population groups of *E. ligea* inhabiting the study area is considerably weaker: (i) they are recovered as genetically different only by population structure analyses conducted on *E. ligea* alone, excluding *E. euryale* (Figure 2); (ii) their genetic differentiation (F_ST_; Figure 3) and divergence (d_XY_; see Section 3.2) are substantially lower than those between the two *E. euryale* lineages (Figure 3); (iii) recent migration rates indicate substantial gene flow between the two groups (Figure 3); (iv) they are not recovered as monophyletic by the phylogenetic analyses (Figure 2); (v) males of the two groups do not differ significantly in the characters of wings and copulatory structures (Figure 4).

Our findings in *E. euryale* are consistent with previous, broader‐scale studies on the variation of mitochondrial COI sequences, wing pattern and male genital morphology across the south‐eastern Alps (Cupedo 2010, 2014; Cupedo and Doorenweerd 2022). On the other hand, our study provides the first phylogeographic assessment within *E. ligea* across the south‐eastern Alps.

The very different levels of differentiation in *E. euryale* and *E. ligea* may be explained by interspecific differences in biological traits and/or colonisation histories. However, as the two species are very similar in many ecological, behavioural and life history features and they are also expected to share other major biological traits as they are strictly related (see Section 2.1), a major role of biological differences may be reasonably ruled out. Therefore, a likely explanation may invoke different colonisation histories. A simple hypothesis is that the two well separate lineages recognised as *E. e. kunzi* and *E. e. ocellaris* diverged over a longer period, in separate regions, possibly outside the study area, and subsequentially colonised this area through distinct routes. Indeed, available phenotypic and molecular data indicate that other populations belonging to the lineage of *E. e. kunzi* occupy the mountain regions west and south of the study area, while other populations belonging to *E. e. ocellaris* also occur to the north and east (Cupedo 2010; Cupedo and Doorenweerd 2022). Therefore, current distributions are consistent with the possibility that the two lineages persisted in separate refugia within the south‐eastern Alps during recent glacial periods (as already hypothesised by Cupedo and Doorenweerd 2022) and later recolonized the Southern Dolomites from different directions (*E. e. kunzi* from the west or south, *E. e. ocellaris* from the north or east). Conversely, we hypothesise that the shallower differentiation between the two population groups of *E. ligea* originated more recently, possibly in situ in our study area, but it is difficult to speculate on the evolutionary and colonisation history of *E. ligea*, given the limited available data from other regions.

### 4.2 Methodological Options and Limitations of the Study

In order to provide compelling evidence for pseudo‐concordance between *E. euryale and E. ligea* within the remarkably heterogeneous mountain region here investigated, we (i) applied a fine scale sampling and (ii) combined genomic and phenotypic analyses.

Sensible phylogeographic investigations require sampling populations at a spatial resolution calibrated to the dispersal abilities of the studied organisms and to the density of potential barriers of the studied region (see Section 1). Therefore, we aimed at sampling specimens of *E. euryale and E. ligea* from locations only few km apart, which approximately matches the expected dispersal abilities of these butterflies (see Section 2.1) and the geographical density of valleys and ridges in the study area (see Figure 1).

However, maximising the sampling density of the study has constrained the extent of the study area, and the trade‐off has hindered the possibility to assess pseudo‐concordance on a broader region.

Moreover, despite our efforts to sample populations uniformly across the study area, remarkably steep slopes and scarcity of accessible gorges hindered our possibilities to adequately explore the Monti del Sole massif, which turned out the zone where eastern and western population groups replace each other in both species (Figure 1). As a consequence, it remains unknown whether the Monti del Sole massif harbours additional populations of *E. euryale* or *E. ligea* and, if so, what the genetic composition of those populations might be. Therefore, it remains to be assessed whether the correspondence of the barrier identified for the two species within this zone would hold at an even finer geographic scale. Furthermore, even though no admixture was found between the populations of *E. e. kunzi* sampled west of Monti del Sole and those of *E. e. ocellaris* sampled east, we cannot rule out the possibility of an admixed intermediate population in between. Further exploration and sampling in this key zone will therefore be essential for determining the exact geographic pattern of lineage replacement and assessing whether the apparent lack of admixture reflects a true barrier or simply a sampling artefact.

Such limitations in spatial extent and resolution also call for some caution in extrapolating our results on the level of differentiation between *E. e. kunzi* and *E. e. ocellaris* beyond the studied area. Even though the populations sampled in our study area show strong evidence of species‐level differentiation (see Section 4.1), a thorough assessment of species boundaries within *E. euryale* as currently circumscribed (also addressing the taxonomic rank of *E. e. kunzi* and *E. e. ocellaris*) will require more extensive sampling across the remaining parts of its range. Such sampling should include other populations belonging to the same subspecies, other subspecies closely related to either *E. e. kunzi* (e.g., *E. e. adyte*, west of our study area) or *E. e. ocellaris* (e.g., *E. e. isarica*, north of our study area; Cupedo and Doorenweerd 2022), as well as other contact or transitional zones between subspecies. In particular, other contact zones between *E. e. kunzi* and *E. e. ocellaris* are known outside of our study area (Cupedo 2014), but so far they have not been investigated by genetic or genomic methods.

Nonetheless, the potential species‐level divergence between *E. e. kunzi* and *E. e. ocellaris* does not compromise our comparative assessment of differentiation patterns in *E. euryale* and *E. ligea*, as all morphological and molecular data indicate that *E. e. kunzi* and *E. e. ocellaris* belong to a single evolutionary lineage nested within *E. euryale* (i.e., they separated from each other only after the split between *E. euryale* and *E. ligea*; Cupedo 2010; Cupedo and Doorenweerd 2022; also our own analyses).

As evolutionary divergence is understood to proceed not only at the genetic level, but also to affect other features (e.g., morphological, ecological and physiological), which may not always show consistent patterns (De Queiroz 2007; Schlick‐Steiner et al. 2010), we both estimated the differentiation among populations both at the genomic and phenotypic levels. For the former, we leveraged thousands of loci sampled across the genome, providing robust insights into population structure, magnitude of gene flow and phylogenetic relationships within both species (McCormack et al. 2013; Edwards et al. 2021). For the latter, we analysed a suite of morphological characters, describing size and colour pattern of the wings, and size, shape and fine structural details of the male copulatory structures. All these traits are often implicated in evolutionary divergence in butterflies and are frequently associated with prezygotic reproductive barriers (e.g., Kronforst et al. 2006; Masly 2012; Mérot et al. 2017). Corroborating molecular analyses with morphological analyses has allowed us to strengthen our case for pseudo‐concordance, as we got evidence that the geographic patterns of differentiation observed at the genomic and phenotypic levels are fully consistent. However, other biological properties remain to be investigated among those frequently or potentially involved in the geographical differentiation among populations, like ecological niche and life history traits.

## 5 Conclusions

By applying a dense spatial sampling and extending our analyses beyond genomic data to include phenotypic features, we obtained compelling evidence for pseudo‐concordance between two closely related butterfly species in the fine‐scale patterns of evolutionary differentiation in a heterogeneous region. The combination of these two methodological options is rare in comparative phylogeography research, yet our case‐study suggests that integrating them can substantially improve our ability to detect and estimate the frequency of occurrence of pseudo‐concordance in natural systems, especially in heterogeneous landscapes.

## Author Contributions

**Luca Gregnanin:** conceptualization (equal), data curation (equal), formal analysis (lead), investigation (equal), resources (equal), visualization (equal), writing – original draft (lead), writing – review and editing (lead). **Enrico Carta:** conceptualization (supporting), investigation (equal), resources (equal), writing – review and editing (supporting). **Claire Dainelli:** investigation (equal), writing – review and editing (supporting). **Roberto Magnolini:** conceptualization (equal), data curation (supporting), investigation (equal), resources (equal), writing – review and editing (supporting). **Giulio Montanaro:** data curation (supporting), investigation (equal), writing – review and editing (supporting). **Alessandro Grapputo:** formal analysis (supporting), funding acquisition (equal), supervision (supporting), writing – review and editing (supporting). **Lucio Bonato:** conceptualization (equal), data curation (supporting), funding acquisition (equal), investigation (equal), resources (equal), supervision (lead), visualization (equal), writing – original draft (supporting), writing – review and editing (lead).

## Funding

This research was supported by the Italian Ministry of University and Research (project funded by the European Union—Next Generation EU: PNRR Missione 4 Componente 2, ‘Dalla ricerca all'impresa’, Investimento 1.4, Progetto CN00000033). This work was also supported by Dolomiti Bellunesi National Park.

## Conflicts of Interest

The authors declare no conflicts of interest.

## Data Availability

The raw reads from ddRAD sequencing of the specimens included in the analyses are available in SRA NCBI database (accession RJNA1358982). The raw morphological data and the R script used for the analysis are available in Dryad (https://doi.org/10.5061/dryad.h9w0vt4xv).

## Appendix A

### Protocol for Taking Photographs of Male Copulatory Structures

To standardise the position of the dorsal copulatory structures (Figure A2A) and of the right valve (Figure A2B), each structure was fully immersed in a dense mixture of EtOH and polyethylene glycol on a microscope slide and covered with a thin layer of 70% EtOH to avoid sparkles. The dorsal copulatory structures (Figure A2A) were oriented with the lateral right side facing upwards, ensuring that the two brachia were fully overlapping; the tip of the uncus, the tip of the right brachium and the notch between the uncus and the right brachium were aligned on the same horizontal focal plane (asterisks in Figure A2A). The right valve (Figure A2B) was positioned with the external side facing upward. The entire dorsal margin of the valve, from the most anterior to the most posterior tips (asterisks in Figure A2B), was aligned on the same horizontal focal plane, including the dorsal anterior projection, the edge of the shoulder and the teeth (Figure A2B).

**TABLE A1:** Specimens examined for the ddRAD molecular data and the phenotypic characters of the wings and the copulatory structures. Coordinates are reported in WGS84 CRS. In ‘ddRAD’: (a) Indicates specimens used for the optimization of parameters of the *de novo* assembly. Specimens are ordered for species and main population group and then W to E.

Specimen ID	Species/subspecies/Genetic group	Date	Region	Locality	Latitude (°N)	Longitude (°E)	Altitude (m)	Sex	N ddRAD reads (*10^6^)	ddRAD	Wings	Copulatory structures
PD‐R 981	*Erebia euryale kunzi*	10/07/2023	Southern Dolomites	M. Masieron: E slope: plot B	46.077	11.841	1550	M	10.74	−	−	−
PD‐R 975	*Erebia euryale kunzi*	10/07/2023	Southern Dolomites	M. Masieron: E slope: plot C	46.080	11.844	1670	M	8.61	+	+	+
PD‐R 976	*Erebia euryale kunzi*	10/07/2023	Southern Dolomites	M. Masieron: E slope: plot C	46.080	11.844	1670	M	6.12	+	+	+
PD‐R 977	*Erebia euryale kunzi*	10/07/2023	Southern Dolomites	M. Masieron: E slope: plot C	46.080	11.844	1670	M	8.28	+ (a)	+	+
PD‐R 978	*Erebia euryale kunzi*	10/07/2023	Southern Dolomites	M. Masieron: E slope: plot C	46.080	11.844	1670	M	8.24	+ (a)	+	+
PD‐R 979	*Erebia euryale kunzi*	10/07/2023	Southern Dolomites	M. Masieron: E slope: plot C	46.080	11.844	1670	M	10.27	+	+	+
PD‐R 982	*Erebia euryale kunzi*	03/07/2023	Southern Dolomites	M. Masieron: E slope: plot C	46.080	11.844	1660	M	4.31	+	+	+
PD‐R 985	*Erebia euryale kunzi*	26/06/2023	Southern Dolomites	V. Fraina: plot A	46.109	11.931	680	M	2.93	+	+	+
PD‐R 916	*Erebia euryale kunzi*	26/07/2022	Southern Dolomites	C. Campedel—C. Pinea: plot D	46.147	11.968	1500	F	2.33	+	−	−
PD‐R 912	*Erebia euryale kunzi*	28/07/2022	Southern Dolomites	C. Campedel—C. Pinea: plot E	46.145	11.969	1530	F	7.63	+ (a)	−	−
PD‐R 914	*Erebia euryale kunzi*	28/07/2022	Southern Dolomites	C. Campedel—C. Pinea: plot E	46.145	11.969	1530	F	7.71	+ (a)	−	−
PD‐R 946	*Erebia euryale kunzi*	28/07/2022	Southern Dolomites	C. Campedel—C. Pinea: plot F	46.143	11.972	1620	M	8.81	+	+	+
PD‐R 913	*Erebia euryale kunzi*	28/07/2022	Southern Dolomites	C. Campedel—C. Pinea: plot C	46.147	11.974	1640	F	9.92	+	−	−
PD‐R 923	*Erebia euryale kunzi*	28/07/2022	Southern Dolomites	C. Campedel—C. Pinea: plot C	46.147	11.974	1640	F	0.28	−	−	−
PD‐R 924	*Erebia euryale kunzi*	27/07/2022	Southern Dolomites	V. di Campotorondo: plot B	46.174	11.991	1760	F	0.58	−	−	−
PD‐R 929	*Erebia euryale kunzi*	27/07/2022	Southern Dolomites	V. di Campotorondo: plot E	46.186	11.991	1480	F	0.04	−	−	−
PD‐R 930	*Erebia euryale kunzi*	27/07/2022	Southern Dolomites	V. di Campotorondo: plot B	46.174	11.991	1760	F	0.28	−	−	−
PD‐R 980	*Erebia euryale kunzi*	27/07/2022	Southern Dolomites	V. di Campotorondo: plot E	46.186	11.991	1480	M	2.44	+	+	−
PD‐R 984	*Erebia euryale kunzi*	27/07/2022	Southern Dolomites	V. di Campotorondo: plot B	46.175	11.992	1750	M	4.93	+	+	+
PD‐R 927	*Erebia euryale kunzi*	27/07/2022	Southern Dolomites	V. di Campotorondo: plot A	46.171	11.993	1820	F	0.42	−	−	−
PD‐R 915	*Erebia euryale kunzi*	27/07/2022	Southern Dolomites	V. di Campotorondo: plot D	46.179	11.994	1620	F	1.26	+	−	−
PD‐R 920	*Erebia euryale kunzi*	27/07/2022	Southern Dolomites	V. di Campotorondo: plot C	46.177	11.994	1650	F	0.02	−	−	−
PD‐R 922	*Erebia euryale kunzi*	27/07/2022	Southern Dolomites	V. di Campotorondo: plot A	46.171	11.994	1820	F	0.57	−	−	−
PD‐R 925	*Erebia euryale kunzi*	27/07/2022	Southern Dolomites	V. di Campotorondo: plot C	46.176	11.994	1700	F	0.03	−	−	−
PD‐R 926	*Erebia euryale kunzi*	27/07/2022	Southern Dolomites	V. di Campotorondo: plot F	46.181	11.994	1560	F	0.07	−	−	−
PD‐R 986	*Erebia euryale kunzi*	27/07/2022	Southern Dolomites	V. di Campotorondo: plot C	46.177	11.994	1650	M	0.92	−	−	−
PD‐R 989	*Erebia euryale kunzi*	18/07/2023	Southern Dolomites	V. de l'Art: plot A	46.217	12.189	1240	M	0.07	−	−	−
PD‐R 983	*Erebia euryale kunzi*	08/07/2023	Venetian Prealps	Venal di Funès: plot A	46.197	12.422	1240	M	6.49	+ (a)	−	−
PD‐R 988	*Erebia euryale kunzi*	08/07/2023	Venetian Prealps	Venal di Funès: plot A	46.197	12.422	1240	M	0.92	−	−	−
PD‐R 957	*Erebia euryale ocellaris*	25/06/2022	Southern Dolomites	V. Vescovà: plot B	46.243	12.158	1240	M	4.43	+	+	+
PD‐R 965	*Erebia euryale ocellaris*	25/06/2022	Southern Dolomites	V. Vescovà: plot B	46.243	12.158	1240	M	2.2	+	+	+
PD‐R 963	*Erebia euryale ocellaris*	19/07/2023	Southern Dolomites	V. de l'Art: plot B	46.223	12.192	1550	M	5.97	+	+	+
PD‐R 962	*Erebia euryale ocellaris*	19/07/2023	Southern Dolomites	V. de l'Art: plot C	46.222	12.199	1770	M	6.39	+ (a)	+	+
PD‐R 959	*Erebia euryale ocellaris*	11/07/2023	Northern Dolomites	S. Vito di Cadore: Tambres	46.464	12.217	1160	M	8.63	+ (a)	−	−
PD‐R 966	*Erebia euryale ocellaris*	04/07/2022	Southern Dolomites	Cajada: plot A	46.239	12.239	1210	M	1.59	+	+	+
PD‐R 958	*Erebia euryale ocellaris*	11/07/2023	Southern Dolomites	P. Cibiana	46.373	12.259	1540	M	3.92	+	+	+
PD‐R 961	*Erebia euryale ocellaris*	16/07/2023	Carnic Alps	Pian di Mazzes: plot A	46.668	12.423	1800	M	6.08	+ (a)	−	−
PD‐R 960	*Erebia euryale ocellaris*	15/07/2023	Carnic Alps	Pian di Mazzes: plot B	46.662	12.428	1760	M	4.65	+	−	−
PD‐R 964	*Erebia euryale ocellaris*	29/07/2023	Carnic Prealps	M. Ciaurlec	46.231	12.831	1080	M	5.36	+ (a)	−	−
PD‐R 971	*Erebia euryale pseudoadyte*	19/07/2023	Mt. Baldo	Cima Valdritta: E slope	45.724	10.855	1610	M	3.52	+	−	−
PD‐R 972	*Erebia euryale pseudoadyte*	19/07/2023	Mt. Baldo	Cima Valdritta: E slope	45.724	10.855	1610	M	5.51	+	−	−
PD‐R 973	*Erebia euryale pseudoadyte*	19/07/2023	Mt. Baldo	Pra Alpesina	45.751	10.864	1900	M	7.69	+ (a)	−	−
PD‐R 974	*Erebia euryale pseudoadyte*	19/07/2023	Mt. Baldo	Pra Alpesina	45.751	10.864	1900	M	8.54	+	−	−
PD‐R 967	*Erebia euryale pseudoadyte*	18/07/2023	Mt. Baldo	Cima delle Redutte: W slope	45.713	10.867	1480	M	5	+	−	−
PD‐R 968	*Erebia euryale pseudoadyte*	18/07/2023	Mt. Baldo	Cima delle Redutte: W slope	45.713	10.867	1500	M	5.66	+	−	−
PD‐R 969	*Erebia euryale pseudoadyte*	18/07/2023	Mt. Baldo	Cima delle Redutte: N slope	45.718	10.872	1560	M	5.27	+	−	−
PD‐R 970	*Erebia euryale pseudoadyte*	18/07/2023	Mt. Baldo	Cima delle Redutte: N slope	45.718	10.872	1560	M	4.29	+	−	−
PD‐R 910	*Erebia ligea* N	14/08/2023	Northern Dolomites	P. Giau	46.487	12.044	2160	F	6.24	+	−	−
PD‐R 918	*Erebia ligea* N	13/08/2023	Southern Dolomites	V. Pramper: plot B	46.321	12.168	1200	F	8.07	+ (a)	−	−
PD‐R 940	*Erebia ligea* W	14/07/2023	Southern Dolomites	M. Grave: E slope	46.092	11.919	1260	M	4.77	+ (a)	+	+
PD‐R 941	*Erebia ligea* W	14/07/2023	Southern Dolomites	M. Grave: S slope	46.091	11.925	1320	M	2.67	+	−	+
PD‐R 949	*Erebia ligea* W	19/06/2023	Southern Dolomites	V. Fraina: plot A	46.108	11.929	690	M	5.41	+	+	+
PD‐R 950	*Erebia ligea* W	19/06/2023	Southern Dolomites	V. Fraina: plot A	46.108	11.929	690	M	5.73	+	+	−
PD‐R 951	*Erebia ligea* W	19/06/2023	Southern Dolomites	V. Fraina: plot A	46.108	11.929	690	M	5.91	+	+	+
PD‐R 952	*Erebia ligea* W	26/06/2023	Southern Dolomites	V. Fraina: plot A	46.109	11.931	680	M	5.55	+	+	−
PD‐R 953	*Erebia ligea* W	19/06/2023	Southern Dolomites	V. Fraina: plot B	46.110	11.934	660	M	8.94	+	+	+
PD‐R 906	*Erebia ligea* W	28/07/2022	Southern Dolomites	Lago della Stua	46.139	11.950	740	F	8.05	+	−	−
PD‐R 904	*Erebia ligea* W	28/07/2022	Southern Dolomites	C. Campedel—C. Pinea: plot A	46.148	11.964	1300	F	6.36	+	−	−
PD‐R 907	*Erebia ligea* W	26/07/2022	Southern Dolomites	C. Campedel—C. Pinea: plot B	46.149	11.966	1470	F	1.19	+	−	−
PD‐R 932	*Erebia ligea* W	26/07/2022	Southern Dolomites	C. Campedel—C. Pinea: plot B	46.149	11.966	1480	M	5.79	+ (a)	+	−
PD‐R 935	*Erebia ligea* E	03/07/2023	Southern Dolomites	V. Imperina: plot A	46.253	12.043	780	M	5.69	+	+	+
PD‐R 954	*Erebia ligea* E	04/07/2023	Southern Dolomites	V. Imperina: plot B	46.248	12.053	1130	M	10.43	+	+	+
PD‐R 955	*Erebia ligea* E	04/07/2023	Southern Dolomites	V. Imperina: plot B	46.248	12.053	1130	M	10.66	+	+	−
PD‐R 956	*Erebia ligea* E	04/07/2023	Southern Dolomites	V. Imperina: plot B	46.248	12.053	1130	M	8.27	+	+	+
PD‐R 931	*Erebia ligea* E	25/06/2022	Southern Dolomites	V. Vescovà: plot A	46.244	12.145	1130	M	7.42	+	+	+
PD‐R 947	*Erebia ligea* E	25/06/2022	Southern Dolomites	V. Vescovà: plot A	46.244	12.145	1130	M	4.51	+ (a)	+	−
PD‐R 909	*Erebia ligea* E	13/08/2023	Southern Dolomites	V. Pramper: plot A	46.325	12.168	1140	F	7.94	+	−	−
PD‐R 933	*Erebia ligea* E	24/06/2022	Southern Dolomites	V. Vescovà: plot C	46.247	12.173	1380	M	2.36	+	+	−
PD‐R 948	*Erebia ligea* E	24/06/2022	Southern Dolomites	V. Vescovà: plot C	46.247	12.173	1380	M	6.49	+	+	+
PD‐R 917	*Erebia ligea* E	03/08/2022	Southern Dolomites	V. dei Ross	46.258	12.185	1170	F	5.11	+	−	−
PD‐R 942	*Erebia ligea* E	19/07/2023	Southern Dolomites	V. de l'Art: plot B	46.223	12.191	1550	M	6.55	+	+	+
PD‐R 939	*Erebia ligea* E	11/07/2023	Northern Dolomites	S. Vito di Cadore: Tambres	46.464	12.217	1160	M	3.39	+ (a)	−	−
PD‐R 905	*Erebia ligea* E	04/07/2022	Southern Dolomites	Cajada: plot B	46.234	12.235	1230	F	4.53	+ (a)	−	−
PD‐R 934	*Erebia ligea* E	04/07/2022	Southern Dolomites	Cajada: plot B	46.234	12.235	1230	M	4.24	+ (a)	+	+
PD‐R 944	*Erebia ligea* E	27/07/2023	Southern Dolomites	Cajada: plot A	46.238	12.238	1200	M	7.02	+	+	+
PD‐R 911	*Erebia ligea* E	04/07/2022	Southern Dolomites	Cajada: plot A	46.239	12.239	1210	F	1.78	+	−	−
PD‐R 945	*Erebia ligea* E	27/07/2023	Southern Dolomites	Cajada: plot C	46.230	12.241	1260	M	4.93	+	+	+
PD‐R 908	*Erebia ligea* E	27/07/2023	Southern Dolomites	Cajada: plot D	46.239	12.243	1180	F	1.08	+	−	−
PD‐R 943	*Erebia ligea* E	27/07/2023	Southern Dolomites	Cajada: plot D	46.239	12.243	1180	M	1.59	+	+	+
PD‐R 936	*Erebia ligea* E	11/07/2023	Southern Dolomites	P. Cibiana	46.373	12.259	1540	M	4.61	+	+	+
PD‐R 937	*Erebia ligea* E	08/07/2023	Venetian Prealps	Venal di Funès: plot B	46.195	12.420	1190	M	3.13	+	−	−
PD‐R 938	*Erebia ligea* E	08/07/2023	Venetian Prealps	Venal di Funès: plot B	46.195	12.420	1190	M	2.96	+	−	−
PD‐R 987	*Erebia ligea*	10/07/2023	Southern Dolomites	M. Masieron:E slope: plot A	46.074	11.832	1370	M	0.02	−	−	−
PD‐R 990	*Erebia ligea*	14/06/2023	Southern Dolomites	V. Fraina: plot B	46.110	11.933	660	M	0.07	−	−	−
PD‐R 921	*Erebia ligea*	26/07/2022	Southern Dolomites	C. Campedel—C. Pinea: plot D	46.146	11.961	1030	F	0.28	−	−	−
PD‐R 919	*Erebia ligea*	26/07/2022	Southern Dolomites	C. Campedel—C. Pinea: plot A	46.148	11.963	1240	F	0.88	−	−	−
PD‐R 1004	*Erebia cassioides*	14/08/2023	Northern Dolomites	P. Giau	46.487	12.045	2150		4.89	+	−	−
PD‐R 1006	*Erebia gorge*	16/07/2023	Carnic Alps	Hornischegg	46.699	12.419	2470		9.45	+	−	−

**TABLE A2:** Morphological characters evaluated on the wings and on the male copulatory structures (see also Figure A2). The terminology for the copulatory structures follows Higgins (1975) and, secondarily, Warren (1936).

Anatomical part and position	Character name	Definition	Character acronym	Value	References	Notes
**Wings**						
Anterior wing: Dorsal side	Size	Length of vein 3 from its origin to the intersection with the margin of the anterior wing	AWL	Measure	Verity 1953	Measured with ruler under microscope
	Androconial patches	Presence of a distinguishable patch of androconial scales on the dorsal side of the anterior wing	AP	Yes/no	Warren 1936; Verity 1953	Evaluated by observing the wing against an intense beam of light
	Size of red patch	Shape and extent of red in the postdiscal area of the dorsal side of the anterior wings	RDAPAS	0–5	Cupedo 2010	0: absent; 1: narrow ring around ocelli, no spot in cell 3; 2: rings drop‐shaped but separated, a spot in cell 3; 3: incomplete band, spots in cell 2 and 3 separate, or spot in cell 3 strongly constricted; 4: band continuous, outer margin concave; 5: band continuous, outer margin straight or convex
	Pupillated ocelli	Presence of at least 1 white scale at the center of an ocellus on the dorsal side of the anterior wings	DAOP	Yes/no	Effinger 1936; Cupedo 2010	
Posterior wing: dorsal side	Pupillated ocelli	Presence of at least 1 white scale at the center of an ocellus on the dorsal side of the posterior wings	DPOP	Yes/no	Cupedo 2010	
	Size of ocelli	The longest cord of the biggest ocellus (considering the border of the black part of the ocellus) on the dorsal side of the posterior wing	DOS	Measure	Verity 1953	Measured with ruler under microscope
	Size of red patch	Shape and extent of red in the postdiscal area of the dorsal side of the posterior wings	RDPPAS	0–3	Cupedo 2010	0: absent; 1: narrow ring around ocelli; 2: spots united at least in cells 4–5‐6, separate in cells 2 and 3; 3: band continuous
Anterior wing: ventral side	Size of red patch	Shape and extent of red in the postdiscal area of the ventral side of the anterior wings	RVAPAS	0–3	Cupedo 2010	0: absent; 1: spots separate, band often incomplete; 2: band complete, strongly constricted or disrupted in cell 3; 3: complete band
	Pupillated ocelli	Presence of at least 1 white scale at the center of an ocellus on the ventral side of the anterior wings	VAOP	Yes/no	Cupedo 2010	
	Abrupt colour transition	Whether the colour changes abruptly between basal and postdiscal areas on the ventral side of the anterior wings	BPT	Yes/no	Verity 1953	Yes: abrupt transition; No: gradual transition or no transition (i.e., same colour in both basal and postdiscal areas)
Posterior wing: ventral side	Size of white patch	Shape of the white patch on the proximal margin of the postdiscal area on the ventral side of the posterior wings	WPS	0–3	Warren 1936; Cupedo 2010	0: absent; 1: white tooth on vein 4; 2: additional white towards costa, even if weak; 3: a continuous white streak from costa to at least vein 4
	Number of ocelli	Number of ocelli (defined as at least three black scale where ocelli are usually present) on the ventral side of the posterior wings	VNO	Count	Warren 1936; Verity 1953	Evaluated under diffuse light
	Size of postdiscal area	Longitudinal length of the light patch in the middle of M3, on the ventral side of the posterior wings	PDS	Measure	Modified from Cupedo (2010)	Measured with ruler under microscope
	Size of ocelli	The longest cord of the ocellus in M3 (considering the border of the black part of the ocellus) on the ventral side of the posterior wings	VOS	Measure	Modified from Cupedo (2010)	Measured with ruler under microscope
	Position of ocelli	Distance between distal margin of the light patch and the centre of the ocellus in M3, on the ventral side of the posterior wings	VOD	Measure	Modified from Cupedo (2010)	Measured with ruler under microscope
	Brown‐red ring around ocelli	Presence of a brown‐red ring at least partially surrounding at least one ocellus, on the ventral side of the posterior wings	BR	Yes/no	Cupedo 2010	
	Relative size of ocelli	VOS/PDS	VORS	Measure	Warren 1936; Verity 1953	
	Relative position of ocelli	VOD/PDS	VOP	Measure	Warren 1936; Verity 1953	
**Copulatory structures**						
Uncus	Length	Distance between the tip of the uncus and the notch between uncus and brachia	UML	Measure	Verity 1953	
	Curvature	Maximum distance of the uncus ventral margin from the line connecting the tip of the uncus and the notch between uncus and brachia	UC	Measure	Verity 1953	
	Height	Width of uncus measured along the perpendicular to the line connecting the tip of the uncus and the notch between uncus and brachia, at its mid‐point	UMH	Measure	Verity 1953	
	Aspect ratio	UML/UMH	UAR	Measure	Warren 1936	
Brachia	Length	Distance between the tip of the right brachium and the notch between uncus and brachia	BL	Measure	Heydemann 1943	
	Height	Width of right brachium measured along the perpendicular to the line connecting the tip of the right brachium and the notch between uncus and brachia, at its mid‐point	BH	Measure	Heydemann 1943	
	Aspect ratio	BL/BH	BAR	Measure	Chapman 1898	
Valve	Length	Distance between the most anterior and the most posterior tips of the valve	VML	Measure	Cupedo 2010	
	Anterior width	width of the valve measured along the perpendicular to the line connecting the anterior and posterior tips of the valve, at 1/3 of the length from the anterior tip	VMAW	Measure	Heydemann 1943	
	Posterior width	Width of the valve measured along the perpendicular to the line connecting the anterior and posterior tips of the valve, at mid‐point between the posterior tip and the perpendicular projection of the most projecting point of the shoulder	VMPW	Measure	Heydemann 1943	
	Aspect ratio	VML/VMAW	VAR	Measure	Warren 1936	
	Narrowing	(VMAW‐VMPW)/VMAW	VN	Measure	Warren 1936	
	Position of 1st tooth	Distance, along the line connecting the anterior and posterior tips of the valve, between the anterior tip of the valve and the perpendicular projection of the tip of the most anterior tooth on the line	FTAP	Measure	Cupedo 2010; Bouaouina et al. 2023	
	Relative position of 1st tooth	FTAP/VML	FTRP	Measure	Dubatolov et al. 1998; Cupedo 2010	Cupedo 2010 calculated ratio between projected distances
	Position of 2nd tooth	Distance, along the line connecting the anterior and posterior tips of the valve, between the anterior tip of the valve and the perpendicular projection of the tip of the second most anterior tooth on the line	STAP	Measure	Cupedo 2010; Bouaouina et al. 2023	
	Relative position of 2nd tooth	STAP/VML	STRP	Measure	Bouaouina et al. 2023	
	Position of 3rd tooth	Distance, along the line connecting the anterior and posterior tips of the valve, between the anterior tip of the valve and the perpendicular projection of the tip of the third most anterior tooth on the line	TTAP	Measure	Bouaouina et al. 2023	
	Relative position of 3rd tooth	TTAP/VML	TTRP	Measure	Bouaouina et al. 2023	
	Maximum length of shoulder teeth	Length of the longest tooth along the posterior border of tooth	VTML	Measure	Cupedo 2010	
	Relative maximum length of shoulder teeth	VTML/VML	TRML	Measure	Cupedo 2010	Cupedo 2010 measured the teeth along their distal slope
	Height of shoulder	Maximum distance between dorsal border of the shoulder (excluding teeth) and the line tangent to the dorsal border of the valve both anteriorly and posteriorly with respect to the shoulder	SH	Measure	Cupedo 2010	
	Width of shoulder	Distance between the two points, along the dorsal border of the valve, that have the maximum curvature and are the closest to maximum projection of the shoulder, one anterior and the other posterior to the shoulder	SW	Measure	Cupedo 2010	
	Shoulder aspect ratio	SH/SW	SAR	Measure	Dubatolov et al. 1998; Cupedo 2010	Cupedo 2010 calculated ratio between projected distances
	Arrangement of teeth	Arrangement of the teeth along the dorsal border of valve	VTR	0–6	Warren 1936	Calculated as the sum of three scores independently given by three authors (lb, lg, gm); each score: 0: teeth variously spaced; 1: teeth possibly arranged in rosettes (one largest tooth surrounded by other smaller teeth); 2: teeth obviously arranged in ‘rosette’.
